# Evaluation of the Association Between Allergic Rhinitis and Middle Ear Dysfunction: A Clinicopathological Study

**DOI:** 10.7759/cureus.40913

**Published:** 2023-06-24

**Authors:** Shaila Sidam, Arshed M P., Ujjawal Khurana, Vikas Gupta, Bhavna Dhingra Bhan

**Affiliations:** 1 Otolaryngology - Head and Neck Surgery, All India Institute of Medical Sciences, Bhopal, Bhopal, IND; 2 Ear Nose Throat, All India Institute of Medical Sciences, Bhopal, Bhopal, IND; 3 Pathology and Laboratory Medicine, All India Institute of Medical Sciences, Bhopal, Bhopal, IND; 4 Otorhinolaryngology, All India Institute of Medical Sciences, Bhopal, Bhopal, IND; 5 Paediatrics, All India Institute of Medical Sciences, Bhopal, Bhopal, IND

**Keywords:** otitis media with effusion, nasal smear, chronic otitis media, allergic rhinitis, absolute eosinophil count (aec)

## Abstract

Introduction: The association between allergic rhinitis and otitis media with effusion (OME) has not yet been well studied in the Indian population. In our study, we have attempted to assess the role of nasal cytology on allergic rhinitis symptomatology and on middle ear dysfunction, and the diagnosis was established by symptomatology, AEC, and a nasal smear for eosinophils.

Material and methods: The present study is a single-centre, hospital-based observational study and was conducted at the Department of Otorhinolaryngology and Head-Neck Surgery (All India Institute of Medical Sciences [AIIMS], Bhopal) among patients with symptoms of allergic rhinitis. The ENT examination included anterior rhinoscopy/diagnostic nasal endoscopy (zero-degree endoscope) along with otoscopy to examine the ear and nose. A nasal smear from the inferior turbinate of the nasal cavity was taken and then examined under a microscope to find out the percentage of eosinophils. The chi-squared test was used for group comparisons of categorical data. Statistical significance was kept at p < 0.05.

Results: In this study, out of 126 subjects with allergic rhinitis, 62 (49.2%) had middle ear dysfunction. The most prevalent middle ear pathologies were eustachian tube dysfunction (ETD; 28 cases, 22%), chronic otitis media (COM [16.6%]), OME (5.5%), and acute otitis media (AOM [4.5%]). ETD made up 45.1% of the ear diseases, with COM (33.87%), OME (11.29%), and AOM (9.6%) following. The mean value of absolute eosinophil count (AEC) among the allergic rhinitis participants (n = 32) was found to be 392.42. Absolute eosinophilia was present in 10 patients out of the 32 subjects being tested. Middle ear pathology was found in 7 of the 10 subjects found positive for absolute eosinophilia. Among the ear pathologies found in the participants (n = 7), the most common were ETD and OME (n = 5).

Conclusion: There was no correlation between nasal smear cytology and the severity of nasal symptoms or middle ear disease. The majority of the participants were in nasal eosinophil grade I and showed sneezing as the most common nasal symptom and COM as the most common middle ear pathologic manifestation.

## Introduction

Allergic rhinitis, also known as hay fever, is a common inflammatory condition of the nasal mucosa due to an increased IgE-mediated immune response to inhalant allergens present in indoor and outdoor environments. It is a global problem, with a prevalence of 10-25% of the world's population, making it one of the top 10 reasons for primary healthcare visits [1,2]. The most common allergens are pollen, house dust mites, pets, and mould.

Allergic rhinitis is part of a spectrum of allergic disorders, including asthma, rhinosinusitis, otitis media, nasal polyposis, allergic conjunctivitis, and recurrent lower respiratory tract infections [3,4]. The condition results in nasal obstruction or congestion, rhinorrhea, nasal itching, and sneezing [5]. Allergy was first defined as an altered state of reactivity to an organic substance called an allergen by Clemens Von Pirquet, a paediatrician, in 1906. The head and neck are the most commonly affected target organs of an allergic reaction, and the effect of allergic reactions on producing nasal symptoms was first reported by Hansel in 1936.

According to the ARIA guidelines of 2001, allergic rhinitis is classified into mild, moderate/severe, intermittent, and persistent [6]. Allergic rhinitis can have a significant negative impact on the quality of life and well-being of patients being seen in otology clinics [7-9].

Nasal cytology is a non-invasive method that can be used to study the changes in nasal mucosa in patients with allergic rhinitis. It involves the examination of the nasal mucosa using a nasal smear to determine the type and number of inflammatory cells present in the mucosa [10]. Nasal cytology can provide valuable information regarding the pathophysiology of allergic rhinitis and its effect on middle ear dysfunction. A study was conducted to assess the role of nasal cytology in allergic rhinitis symptomatology and middle ear dysfunction in Indian patients [11]. The study showed that the patients with allergic rhinitis had a higher number of eosinophils and mast cells in their nasal cytology than the healthy controls. The middle ear function was also significantly impaired in the patients with allergic rhinitis compared to the healthy controls. Thus, nasal cytology can provide valuable information regarding the pathophysiology of allergic rhinitis and its effect on middle ear dysfunction.

Otitis media with effusion (OME) is a condition where fluid accumulates in the middle ear, leading to reduced hearing and sometimes ear pain. Several studies have investigated the association between allergic rhinitis and OME. One study found that children with allergic rhinitis were more likely to develop OME than those without allergic rhinitis and that the severity of OME was correlated with the severity of allergic rhinitis [12]. Another study found that treatment of allergic rhinitis with intranasal corticosteroids reduced the incidence of OME in children [13]. The mechanism behind the association between allergic rhinitis and OME is not fully understood. It has been suggested that the inflammation in the nasal mucosa caused by allergic rhinitis can lead to blockage of the eustachian tube, which connects the middle ear to the back of the throat, and that this can cause fluid to accumulate in the middle ear [14]. However, other studies have suggested that there may be additional factors involved, such as changes in the immune system or alterations in the structure of the Eustachian tube [15]. Overall, while the association between allergic rhinitis and OME is not yet fully understood, several studies have suggested that there is a link between the two conditions. Further research is needed to better understand this relationship and identify effective strategies for preventing and treating OME in individuals with allergic rhinitis.

The risk of developing OME is high with allergic rhinitis, thus making the relationship between allergic rhinitis and OME unclear. The link between allergic rhinitis and OME has not been well studied in the Indian population. The study was undertaken to determine the relationship and determinants between allergic rhinitis and ear pathology, with special reference to otitis media with effusion.

## Materials and methods

This was a single-centre, hospital-based, observational study carried out at the Department of Otorhinolaryngology and Head-Neck Surgery, AIIMS, Bhopal, India, from January 2020 to July 2021. The study commenced after approval of the Institutional Human Ethics Committee (IHEC) vide approval number IHEC/LOP/2019/MD0073. The study included patients aged less than 18 years with symptoms of allergic rhinitis who attended the outpatient department (OPD) of the Department of Otorhinolaryngology and Head-Neck Surgery or the Department of Pediatrics, AIIMS, Bhopal, from January 2020 to July 2021. Patients from peripheral health centers working under AIIMS Bhopal were also included in the study. A sample size of 150 was desired, however, due to the COVID-19 pandemic, a sample size of 126 could be achieved.

Inclusion and exclusion criteria

The inclusion criteria were patients with symptoms of allergic rhinitis aged 5 to 18 years. The exclusion criteria were patients with a history of ear disease before the initiation of symptoms of allergic rhinitis, patients with a history of ear or nasal surgeries, patients suspected to have congenital or acquired immune deficiency, patients with a grossly deviated nasal septum or cleft palate, patients with Down's syndrome, and those who refused to provide consent for the study.

Data collection

The patient information sheet was handed over to all the subjects. A detailed clinical examination was done in the OPD, which included anterior rhinoscopy/diagnostic nasal endoscopy (0° endoscope), otoscopic examination, examination of the throat, and clinical examination of the eye.

In the examination of the nose, pale mucosa, serous discharge, turbinate hypertrophy, nasal polyps, or polypoidal mucosa were considered features of allergic rhinitis. An ear examination was done with the help of an Otoscope (2.5 magnification) to examine the findings of middle ear dysfunction (Eustachian tube dysfunction [ETD], acute otitis media [AOM], otitis media with effusion, and chronic otitis media [COM]) associated with allergic rhinitis, like tympanic membrane congestion, retracted tympanic membrane, amber colour, air-fluid, air bubbles in the middle ear, tympanic membrane perforation, tympanosclerotic patch, and healed tympanic membrane. Tympanometry was performed with a pure-tone audiometer (MAICO MI44) with a Probe tone frequency of 226 Hz. Then tympanograms were classified according to Jerger’s classification, with classes B and C used as diagnoses for otitis media with effusion and eustachian tube dysfunction.

Nasal Smear

A nasal smear from the inferior turbinate of the nasal cavity was taken for cytological analysis. The smear was stained with Wright Giemsa stain, and if two smears were taken, then an alcohol-fixed Pap-stained smear was also prepared. The smear was assessed for a grade of inflammation and a percentage of eosinophils. The grades of inflammation were assessed in a subjective way to assess the amount of inflammation (all types of inflammatory cells) on a low-power field and were categorized as no, mild, moderate, and marked. For eosinophils, a differential of 100 cells was done, and the percentage of eosinophils was derived by the manual finger counter method. Scoring for nasal smear eosinophils: grade 1: <5% eosinophil count; grade 2: 5-15% eosinophil count; grade 3: 16-25% eosinophil count; grade 4: >25% eosinophil count.

Absolute Eosinophil Count

The blood sample for the absolute eosinophil count was collected in an EDTA vial. The samples were run on an automated blood cell counter, and the results were expressed in several eosinophils/µL. An absolute eosinophil count greater than or equal to 420/µl was considered blood eosinophilia.

Sample size calculation

A sample size of at least 150 was required, but due to the COVID-19 epidemic and national lockdown during the study period, the study participants were reduced to 126. This sample size approximates a normal distribution with a power of 80% within a confidence interval of 95%.

Statistical analysis

The collected data were coded and recorded in the MS Excel spreadsheet programme (Microsoft® Corp., Redmond, WA). SPSS v23 (IBM Corp., Armonk, NY) was used for data analysis. Descriptive statistics were elaborated in the form of means/standard deviations, medians, and IQRs for continuous variables and frequencies and percentages for categorical variables. Data were presented graphically wherever appropriate for data visualization using column and bar charts for continuous and categorical data, respectively. Group comparisons were made using Wilcox’s test for continuous data and Fisher’s exact test for categorical data when comparing two groups. Statistical significance was kept at p < 0.05.

Ethical considerations

The study was approved by the institutional human ethics committee at AIIMS, Bhopal. An informed consent/assent form was obtained from all participants or their legal guardians. The data collected were kept confidential, and the privacy of the participants was maintained throughout the study.

## Results

A total of 126 subjects with allergic rhinitis participated in this study. The mean age of the participants was 11.2 ± 3.74 years, with the youngest participant being five years old and the eldest being 18 years old. The male-to-female ratio was 2.23:1, with 87 (68.8%) participants being male and 39 (31.2%) participants being female. The maximum number of participants belonged to the age group of 9-12 years.

**Figure 1:**
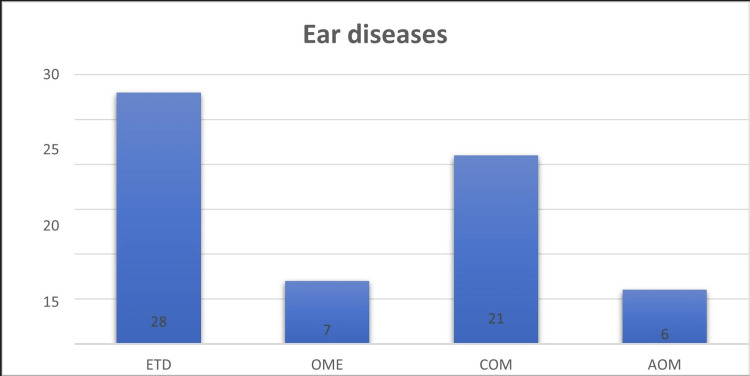
Frequency distribution showing various ear diseases associated with allergic rhinitis (n=62)

**Figure 2:**
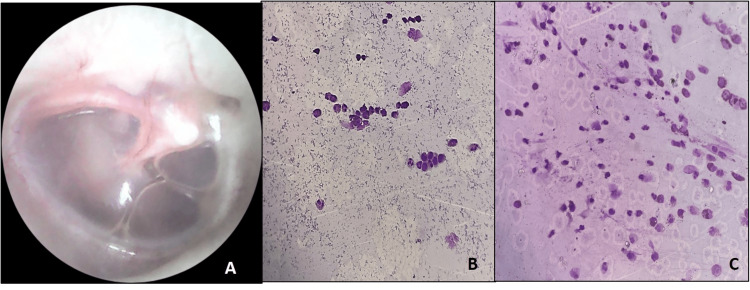
(A) Otoscopic examination of serous otitis media; (B) nasal smear showing moderate grade inflammation with 38% eosinophils (Wright Giemsa; ×400); (C) nasal smear showing moderate grade inflammation with 38% eosinophils (Wright Giemsa; ×400)

Middle ear dysfunction was present in 62 subjects (49.2%) of the total population. Eustachian tube dysfunction was the most common ear disease, present in 28 cases (22%). Other ear diseases observed were COM in 21 cases (16.6%), OME in 7 cases (5.5%), and AOM in 6 cases (4.7%). The combination of Eustachian tube dysfunction and OME accounted for 35 cases (27.78%) out of the total of 126 allergic rhinitis cases (Figures 1-2).

Nasal smear cytology was done for 115 cases of allergic rhinitis. Most patients had some grade of inflammation (106/115), with grade 1+ inflammation (41/115) being the most frequent, followed by grade 2+ (38/115) and grade 3+ in 27/115 cases. The patients presented with various symptoms like nasal blockage, runny nose, itching, and sneezing (Table 1).

**Table 1:** Distribution of nasal symptoms with grade of inflammation of nasal smear

Grade of inflammation	Nasal blockage		Runny nose		Itching		Sneezing	
	Yes	No	Yes	No	Yes	No	Yes	No
0 (no)	9	0	8	1	7	2	9	0
1+ (mild)	33	7	31	9	34	6	39	1
2+ (moderate)	32	6	36	2	27	11	38	0
3+ (marked)	24	4	24	4	26	2	27	1

The nasal eosinophil percentage ranged from 0% to 82% with grade 4. Grade 1 eosinophil (eosinophil <5%) was present more commonly, with 64 participants showing this grade (64/115 cases). Grade 2 eosinophils (eosinophils 6-15%) were present in 23 participants (23/115), and grades 3 (eosinophils 15-25%) and 4 (eosinophils >25%) were present in 13 and 15 participants, respectively.

The absolute eosinophil count (AEC) ranged from 50 to 1261, with a mean of 392.42. The standard deviation was 332, and the median was 280, with an interquartile range (IQR) of 45-525.

Absolute eosinophilia was present in 10 patients out of the 32 subjects being tested. Middle ear pathology was found in 7 out of the 10 subjects found positive for absolute eosinophilia. Among the ear pathologies found in the participants (n = 7), the most common were Eustachian tube dysfunction and OME (n = 5).

## Discussion

In this indexed study, the authors evaluated the prevalence of various middle ear pathologies associated with allergic rhinitis. Out of 126 participants, 62 subjects (49.2%) had middle ear pathology, with the most common being eustachian tube dysfunction (45.1%), followed by COM (33.87%), OME (11.29%), and AOM (9.6%). It was evident from the study that allergic rhinitis leads to eustachian tube dysfunction, which is the main common gateway responsible for the initiation of various middle ear pathologies [1]. The result is similar to the study by Norhafiza et al. to find the prevalence of allergic rhinitis in patients with otitis media with effusion. The conclusion was that allergic rhinitis is a common risk factor for the development of otitis media with effusion. [16]

Allergic rhinitis is characterized by one or more symptoms that include sneezing, itching, nasal congestion, and rhinorrhea. Mucosal inflammation in allergic asthma and rhinitis is characterized by tissue eosinophilia [2]. This was also seen in the study by Ravi et al., which depicts nasal smear eosinophilia as more prevalent in allergic rhinitis [17].

Most participants had some grade of inflammation, of which grade 1 was the most common. Eustachian tube dysfunction and sneezing were commonly seen in participants with grade 1 inflammation. There was no correlation found between the severity of nasal symptoms and middle ear pathology with increasing grade of inflammation. The result is similar to the study by Indranil et al., which concluded that nasal smear eosinophils are a poor indicator of the degree, severity, and duration of allergic rhinitis [18].

Nasal smear eosinophilic count was classified into four grades and compared with ear pathologies and nasal symptoms. Grade 1 eosinophilia was found more often in the participants with allergic rhinitis, with sneezing and COM being the most common manifestations associated with it, followed by ETD.

There was no correlation found between the severity of nasal symptoms and middle ear pathology and an increase in nasal eosinophilic grade. From the above findings, it is evident that around 50% of participants with allergic rhinitis had a middle ear pathological condition associated with it. Among the middle ear pathologies, the most common was Eustachian tube dysfunction (22%), followed by COM (16.6%), OME (5.5%), and AOM (4.5%). Otitis media with effusion was seen in seven cases (5.5%) out of 62 subjects with middle ear pathology. There was no correlation found between nasal cytology and the severity of nasal symptoms or middle ear dysfunction.

In this study, the absolute eosinophil count (AEC) was done on 32 subjects out of 126 participants. The mean value of AEC among the allergic rhinitis participants (n = 32) was found to be 392.42, and absolute eosinophilia was present in 10 patients out of the 32 subjects being tested. Middle ear pathology was found in 7 of the 10 subjects found positive for absolute eosinophilia. Among the ear pathologies found in the participants (n = 7), the most common were ETD and OME (n = 5).

These patients are treated with various modalities like antiallergic medications, steroidal nasal spray, and the Valsalva manoeuvre.

Limitations of the study

The study was performed in the paediatric population, and there was apprehension about blood sampling for AEC counts. The other limitation was that due to restrictions during the COVID-19 pandemic, the sample size got reduced due to the non-availability of regular OPD services. The presentation of allergic rhinitis symptomatology also overlapped with COVID-19 symptoms, and this further added to the limitations that tympanometry could not be performed in all the patients.

## Conclusions

This study highlights the association of allergic rhinitis with middle ear pathology and emphasizes the need to treat allergic rhinitis to prevent middle ear pathologies, which can cause hearing loss for a long duration and affect the psychosocial growth of the child. Particular attention should be given to the function of the eustachian tube to reduce middle ear pathologies. Exercises like the Valsalva manoeuvre should be encouraged during the treatment to reduce the chances of the spread of a middle ear infection.

A holistic approach involving clinical examination, tympanometry, audiometry, nasal smear examination, AEC counts, IgE levels, and skin prick tests, along with medical treatment for complete resolution, is advocated for managing paediatric allergic rhinitis patients.
